# Supplementary material for: Oxytocin facilitates social behavior of female rats via selective modulation of interneurons in the medial prefrontal cortex
Source: Nat Commun. 2026 Feb 20;17:1932. doi: 10.1038/s41467-026-68347-x (PMC12923783; doi:10.1038/s41467-026-68347-x)

SUPPLEMETARY INFORMATION

Oxytocin facilitates social behavior of female rats via selective modulation of interneurons in the medial prefrontal cortex

Schimmer, Kania, Lefevre, et al.

Supplementary Information included:

I. Supplementary Figures
II. Original uncut Western blot shown in Supplemental Figure S3 B2

SUPPLEMENTARY FIGURES

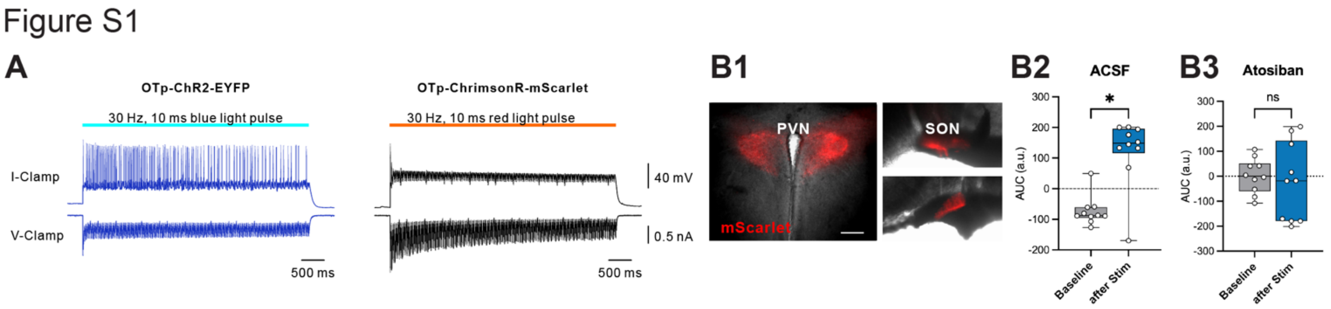

Supplementary Figure S1.
A Functional verification of OTp-ChR2 and OTp-ChrimsonR with *ex vivo* patch-clamp recordings.
B1 Representative OTp-ChrimsonR-mScarlet expression in the PVN and SON. **B2** AUC for OT-sensor signal before and after stimulation of OT fibers in the ILC with red light. **B3** AUC for OT-sensor signal before and after stimulation of OT fibers in the ILC with red light after bath application of an OTR-antagonist (Atosiban) (n=10 sections).

Statistical significance is indicated as ∗ p<0.05, ∗∗ p<0.01, ∗∗∗ p<0.001, ∗∗∗∗ p<0.0001. Box plots show the median, 25th-75th percentiles, and whiskers from minimum to maximum. For details on statistical tests please refer to Supplementary Data 1.

Figure S2

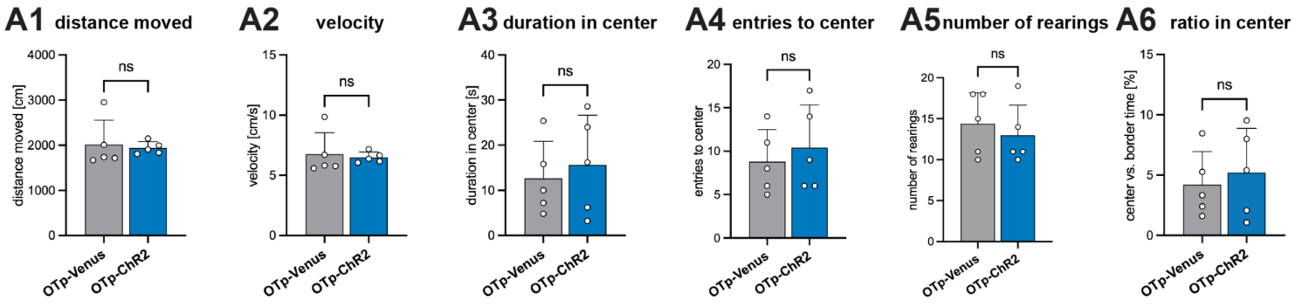

Supplementary Figure S2.

A Exploratory behavior in the open field after optogenetic activation of OT axons in the ILC. **A1** Distance moved and **A2** velocity in the open field, a control for locomotor effects, remain unchanged by optogenetic manipulation of OT axons in the ILC. **A3** Duration in center and **A4** entries to center, a control for anxiety-like behavior, remain unchanged by optogenetic manipulation. **A5** Number of rearings as a non-social behavior is not affected by optogenetic manipulation (n=5 animals per group). **A6** The percentage of time spent in the center of the arena remains unchanged. Error bars show mean±sd. For details on statistical tests please refer to Supplementary Data 1.

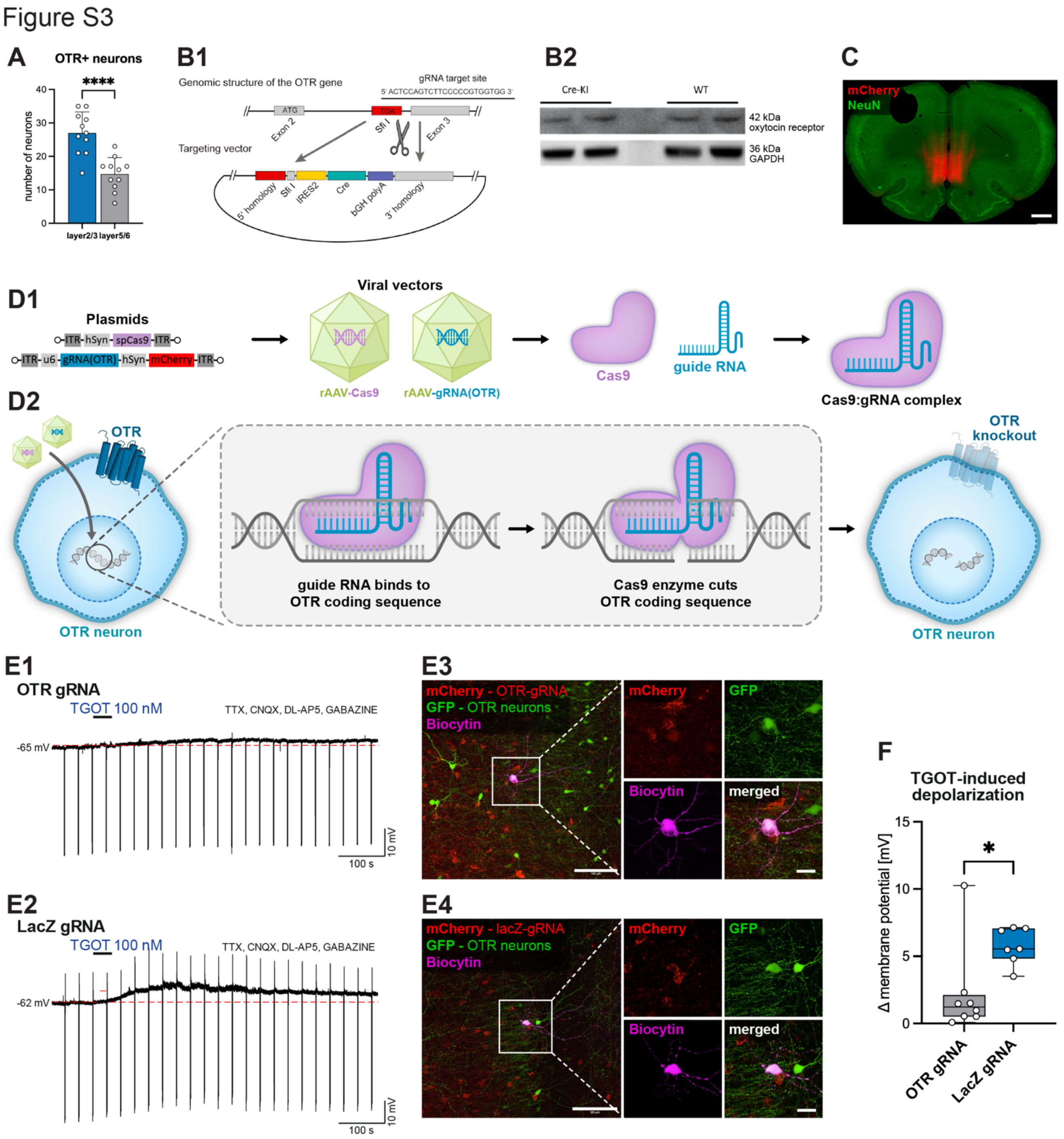

Supplementary Figure S3.

A More OTR+ neurons are found in layer 2/3 of the ILC, compared to layer 5/6.

B1 Generation of the transgenic OTR-Cre rat line utilizing CRISPR/Cas9. **B2** Western blot analysis of the OTR-levels in both transgenic rat line (Cre-KI) and wildtype (WT).

C Injection site of the Cas9/gRNA virus system in the ILC.

D Mechanism of OTR depletion in neurons utilizing a viral CRISPR/Cas9 approach. **D1** Two viral vector plasmids are used to produce rAAVs expressing either the Cas9 enzyme or a specific guide RNA for OTR. **D2** After injection of the mixture of both viruses into the ILC, the Cas9:gRNA complex splices the OTR coding sequence in all neurons and leads to a partial functional OTR knockout, while the neurons themselves remain viable. As a control, a guide RNA for lacZ is used, as no unspecific splicing is expected.

E Representative traces of a whole-cell patch clamp recordings (current clamp, zero current mode) obtained from an OTR+ ILC neuron transfected with AAVs carrying **E1** Cas9 and OTR gRNA or **E2** Cas9 and LacZ gRNA. A TGOT-induced depolarization in the control condition (LacZ) and its absence in the OTR depletion was observed. Downward deflections represent voltage responses of the recorded neuron to hyperpolarizing current injections. **E3** Series of fluorescent projection images of biocytin-filled OTR+ ILC neurons transfected with AAVs carrying Cas9 and OTR gRNA or **E4** Cas9 and LacZ gRNA.

F TGOT-induced depolarization is significantly reduced in the OTR+ ILC neurons transfected with AAVs carrying Cas9 and OTR gRNA (n=8 neurons) compared to the control condition (n=7 neurons).

Statistical significance is indicated as ∗ p<0.05, ∗∗ p<0.01, ∗∗∗ p<0.001, ∗∗∗∗ p<0.0001. Error bars show mean±sd. Box plots show the median, 25th-75th percentiles, and whiskers from minimum to maximum. For details on statistical tests please refer to Supplementary Data 1.

Figure S4

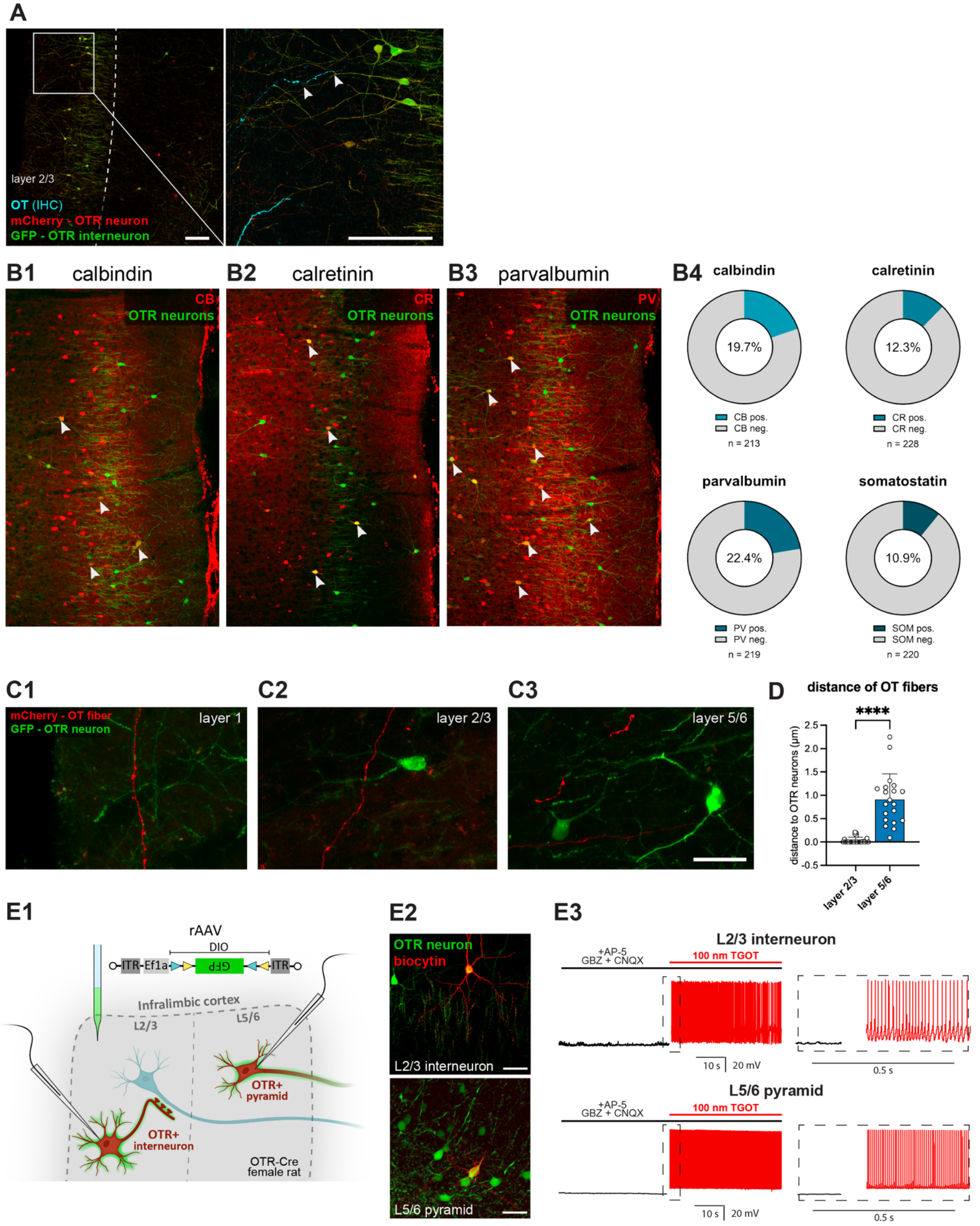

Supplementary Figure S4.

A Alternative representative scan to Figure 4 B2. Scalebar 100µm.

B Heterogenous OTR interneuron population in the ILC. **B1-C3** Representative images of ILC sections previously injected with a Cre-dependent rAAV expressing GFP in OTR neurons and counterstained for various interneuron markers: calbindin (CB), calretinin (CR) and parvalbumin (PV). Scalebar. **B4** Quantitative analysis of the OTR interneuron population; both calbindin or parvalbumin are expressed in about 20% of OTR interneurons in the ILC, while calretinin or somatostatin are found in 10% of interneurons (n=4 animals). Scalebar 150µm.

C OT fibers in close proximity to OTR^+ neurons. OT fibers labelled by viral injection of rAAV-OTp-mCherry in the PVN and SON are found **C1** close to the midline in layer 1, **C2** in close proximity to an OTR^+ interneuron in layer 2/3, and **C3** in close proximity to a pyramidal neuron in layer 5/6. Scalebar 40µm.

D Automated, high-throughput analysis of the distance of OT fibers to OTR neurons in the ILC. Fibers are closer to neurons in layer 2/3 compared to layer 5/6 (n=24 neurons and n=20 neurons, sections obtained from 4 animals).
E Ex vivo electrophysiological characterization of OTR+ neurons in the ILC. **E1** Injection scheme depicting the injection of a rAAV expressing GFP in a Cre-dependent manner in OTR-Cre animals for the identification of OTR+ neurons during ex vivo patch clamp recordings. **E2** Biocytin filled layer 2/3 interneuron and layer 5/6 pyramidal neuron. Scalebar 50μm. **E3** Representative traces of OTR+ neurons firing upon bath application of an OTR agonist (TGOT) (n=9 cells recorded from 4 rats for interneurons, n=5 cells recorded from 2 rats for pyramidal neurons).
Statistical significance is indicated as $*$ $p<0.05$, $**$ $p<0.01$, $***$ $p<0.001$, $****$ $p<0.0001$. Error bars show mean±sd. For details on statistical tests please refer to Supplementary Data 1.

Figure S5

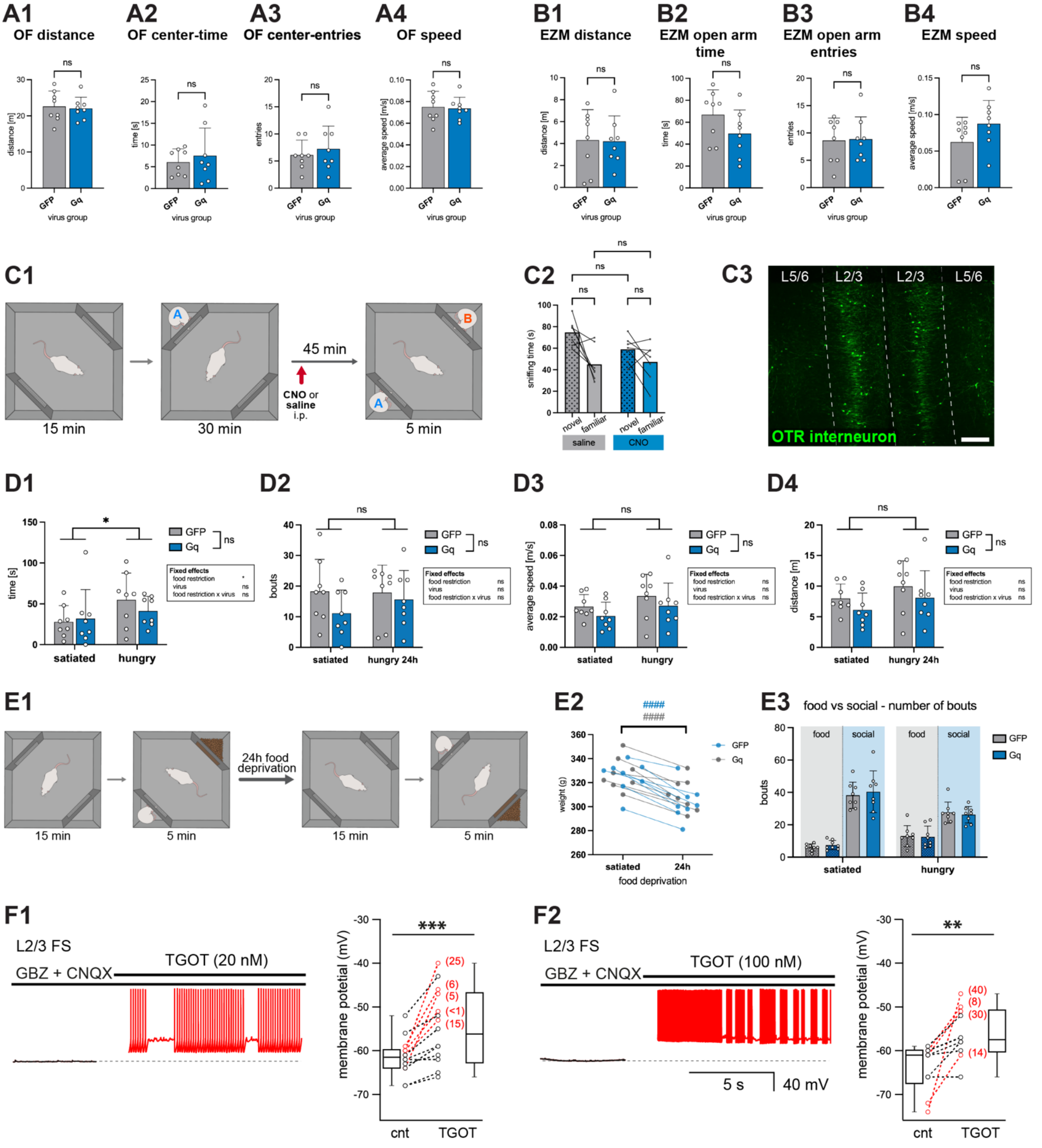

Supplementary Figure S5.

A Exploratory behavior in the open field (OF) after chemogenetic activation of OTR$^+$ interneurons in the ILC. **A1** Distance travelled in the OF, **A2** Time in the center of the OF, **A3** number of entries into the OF, and **A4** average speed in the OF (n=8 animals).

B Exploratory behavior in the elevated zero maze (EZM). **B1** Distance travelled in the EZM, **B2** Time spent in the open parts of the EZM, **B3** number of entries into the open parts of the EZM, and **B4** average speed in the EZM (n=8 animals).

C Social novelty preference paradigm. **C1** Paradigm timeline: animals were habituated to an open field arena with two empty corner compartments for 15 minutes, before placing an unknown female conspecific (animal A) into one corner for a 30 min familiarization session; after a 45 min break, both the familiar (A) and a novel (B) conspecific rat were placed in opposite corners, in order to test social novelty preference. CNO was administered 40 min before the start of the 5 min test session. Corners were placed randomly and altered between sessions and animals. **C2** No effect of CNO administration was observed compared to saline administration (n=7 animals). **C3** Representative injection site of rAAV-DLX-DIO-Gq-GFP for the food vs. social paradigm and social novelty preference paradigm, as verified for all experimental animals post-hoc. Scalebar 500µm.

D Food interest. Animals were placed in an OF with food pellets behind a mesh in one corner. **D1** The time spent investigating the food was only influenced by hunger, but not chemogenetic activation of OTR$^+$ interneurons. **D2** Number of bouts while investigating food in the OF. **D3** Average speed in the OF as well as **D4** distance travelled in the OF (n=8 animals per group).

E Food vs. social behavior paradigm. **E1** Paradigm timeline: animals were habituated to an open field arena with two empty corner compartments for 15 minutes, before placing an unknown female conspecific into one corner, and food into the opposite corner; after 24h of food deprivation the test was repeated. CNO was always injected 40 min before the start of the 5 min choice session. Corners were placed randomly and altered between sessions and animals. **E2** Weight loss of rats after 24h of food deprivation was verified for both groups. **E3** The number of bouts for food and social corners were unaltered (n=8 animals per group).

F Representative traces of whole-cell patch clamp recordings (current clamp, zero current mode) and quantitative analysis of membrane potential changes in layer 2/3 fast-spiking interneurons upon bath application of either **F1** 20nM TGOT (n=14 cells, sections obtained from 4 rats; p=0.0005) or **F2** 100nM TGOT (n=10 cells, sections obtained from 4 rats; p=0.0097).

Statistical significance is indicated as * p<0.05, ** p<0.01, *** p<0.001, **** p<0.0001. Statistical significance of post-hoc tests is indicated as # p<0.05, ## p<0.01, ### p<0.001, #### p<0.0001. Error bars show mean±sd. Box plots show the median, 25th-75th percentiles, and whiskers from minimum to maximum. For details on statistical tests please refer to Supplementary Data 1.

Figure S6

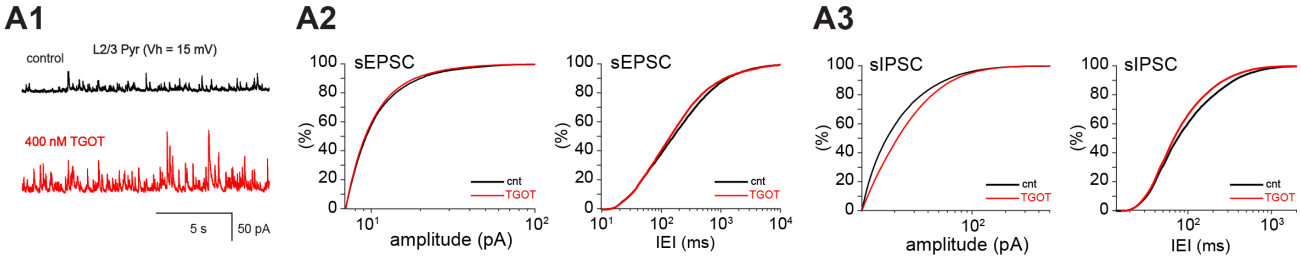

Supplementary Figure S6.

A Analysis of EPSCs (n=37) and IPSCs (n=38) of layer 2/3 OTR negative pyramids after TGOT application (400nM) compared to baseline (cnt). The brain sections were obtained from 10 rats. **A1** Representative trace of IPSC recording. **A2** No effect on EPSC amplitude or kinetics was observed, however **A3** IPSC amplitude (and charge transferred) are increased (20.7 ± 2.3 vs. 23.6 ± 1.8 pA, p = 0.0017; 2173 ± 222 vs. 2657 ± 837 pC, p=0.0013, respectively). IEI: inter-event-interval.

Figure S7

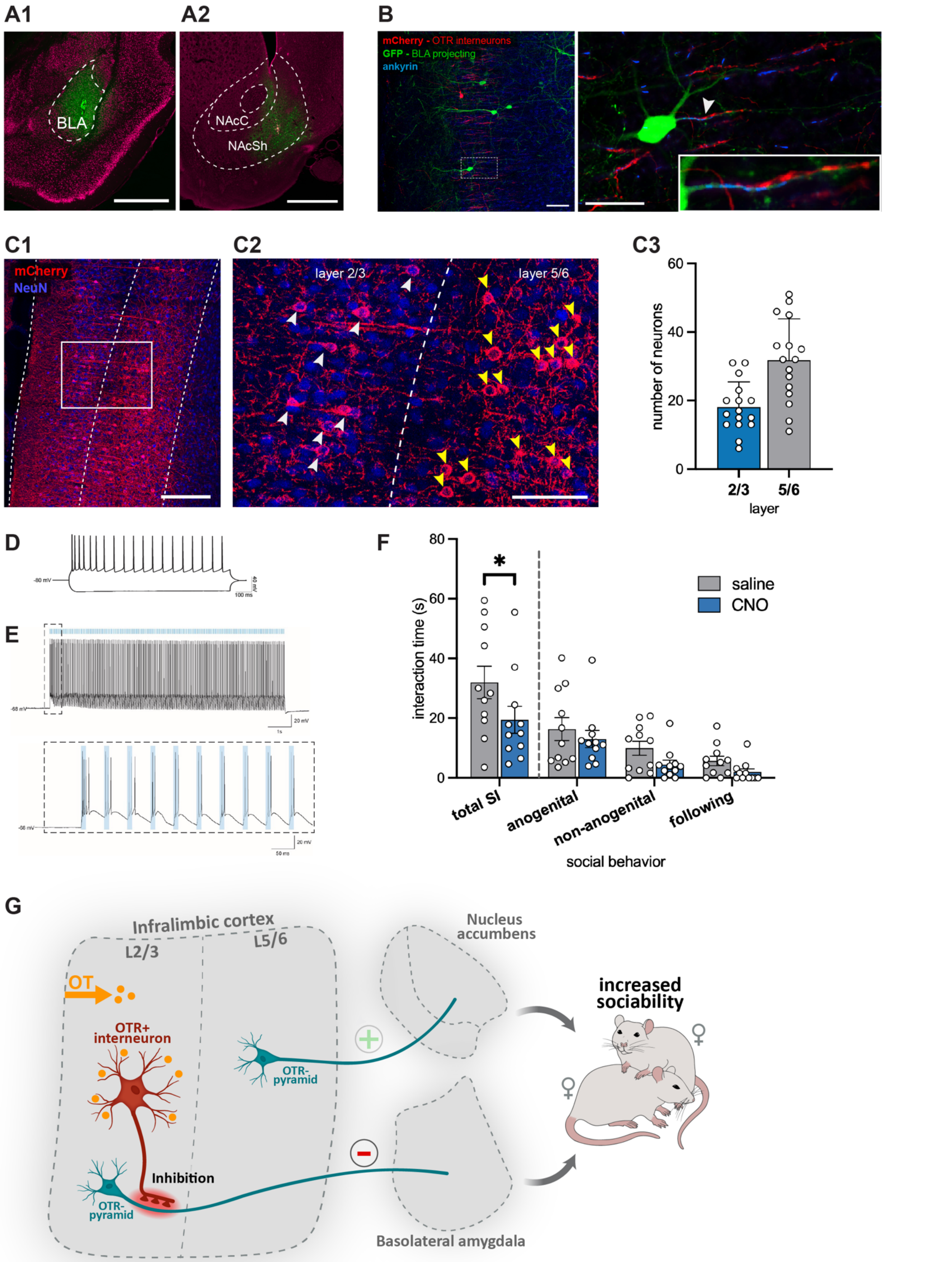

Supplementary Figure S7.

A Representative injection sites of retro-AAV-hSyn-GFP in the **A1** BLA or **A2** NAc. The position of the pipette tip during the injection is marked with retrobeads (white arrow).

B Representative axo-axonic contact (white arrow) of OTR+ interneuron (red) on BLA-projecting pyramidal neuron (green). Scalebar 50µm overview, 25µm zoom.

C Quantification of BLA-projecting neurons in layer 2/3 and layer 5/6 of the ILC. **C1** Overview of the ILC with BLA-projecting neurons in red (rAAV-FRT-Gq-mCherry in ILC, retro-rAAV-Flp in BLA) and NeuN staining (blue). Scalebar 250µm. **C2** BLA-projecting neurons in layer 2/3 (white arrows) and layer 5/6 (yellow arrows). Scalebar 100µm. **C3** Number of BLA-projecting neurons in layer 2/3 and layer 5/6 of the ILC.

D Representative traces of a whole-cell patch clamp recordings (current clamp, zero current mode) obtained from a BLA-projecting ILC neuron showing spike frequency adaptation typical for pyramidal cells.

E Representative traces of a whole-cell patch clamp recordings (current clamp, zero current mode) obtained from an OTR^+ ILC interneuron expressing ChR2 and following to BL stimulation (20Hz, 10ms long pulses, ~10mW). The right trace shows a zoom of the first 10 stimulations.

F Chemogenetic activation of BLA-projecting neurons decreases total social interaction time (n=11 animals). Subbehaviors analyzed were anogenital sniffing, non-anogenital sniffing, and following behavior.

G Working hypothesis. OT projections from the hypothalamus innervate the infralimbic cortex, where they specifically activate OTR^+ interneurons in layer 2/3. These OTR^+ interneurons, which show functional and anatomical properties of Chandelier interneurons, preferentially target and inhibit OTR^- pyramidal neurons projecting to the basolateral amygdala (BLA), decreasing neuronal activity in the BLA. Specific and targeted activation of this OTR^+ neuronal population increases sociability and social preference in female rats.

Statistical significance is indicated as $*$ p<0.05, $**$ p<0.01, $***$ p<0.001, $****$ p<0.0001. Error bars show mean±sd. For details on statistical tests please refer to Supplementary Data 1.

ORIGINAL UNCUT WESTERN BLOT SHOWN IN SUPPLEMENTAL FIGURE S3 B2

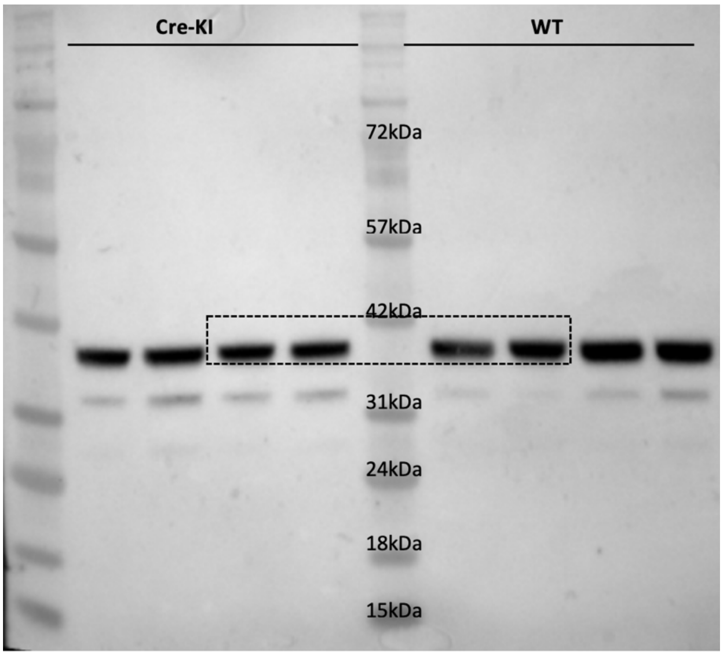

Supplement: Supplementary file 1 — Supplementary Information [file 41467_2026_68347_MOESM1_ESM.pdf]
