# Supplementary material for: Oxytocin facilitates social behavior of female rats via selective modulation of interneurons in the medial prefrontal cortex
Source: Nat Commun. 2026 Feb 20;17:1932. doi: 10.1038/s41467-026-68347-x (PMC12923783; doi:10.1038/s41467-026-68347-x)
Supplement: Supplementary file 2 — Description of Additional Supplementary Files [file 41467_2026_68347_MOESM2_ESM.pdf]

Description of Additional Supplementary Files

File name: Supplementary Data 1

Description: Supplementary Data 1 includes statistical test results and details for all performed tests, divided on individual Excel sheets per Figure. The statistical tests performed with all plotted data in this study have been deposited in the zenodo database under accession code https://doi.org/10.5281/zenodo.17903734.
