# Supplementary material for: Oxytocin facilitates social behavior of female rats via selective modulation of interneurons in the medial prefrontal cortex
Source: Nat Commun. 2026 Feb 20;17:1932. doi: 10.1038/s41467-026-68347-x (PMC12923783; doi:10.1038/s41467-026-68347-x)
Supplement: Supplementary file 4 — Reporting Summary [file 41467_2026_68347_MOESM4_ESM.pdf]

Corresponding author(s): Valery Grinevich

Last updated by author(s): 12.12.2025

Reporting Summary

Nature Portfolio wishes to improve the reproducibility of the work that we publish. This form provides structure for consistency and transparency in reporting. For further information on Nature Portfolio policies, see our Editorial Policies and the Editorial Policy Checklist.

Statistics

For all statistical analyses, confirm that the following items are present in the figure legend, table legend, main text, or Methods section.

n/a	Confirmed	
☐	☒	The exact sample size (*n*) for each experimental group/condition, given as a discrete number and unit of measurement
☐	☒	A statement on whether measurements were taken from distinct samples or whether the same sample was measured repeatedly
☐	☒	The statistical test(s) used AND whether they are one- or two-sided<br>*Only common tests should be described solely by name; describe more complex techniques in the Methods section.*
☐	☒	A description of all covariates tested
☐	☒	A description of any assumptions or corrections, such as tests of normality and adjustment for multiple comparisons
☐	☒	A full description of the statistical parameters including central tendency (e.g. means) or other basic estimates (e.g. regression coefficient) AND variation (e.g. standard deviation) or associated estimates of uncertainty (e.g. confidence intervals)
☐	☒	For null hypothesis testing, the test statistic (e.g. *F*, *t*, *r*) with confidence intervals, effect sizes, degrees of freedom and *P* value noted<br>*Give P values as exact values whenever suitable.*
☒	☐	For Bayesian analysis, information on the choice of priors and Markov chain Monte Carlo settings
☒	☐	For hierarchical and complex designs, identification of the appropriate level for tests and full reporting of outcomes
☒	☐	Estimates of effect sizes (e.g. Cohen's *d*, Pearson's *r*), indicating how they were calculated

Our web collection on statistics for biologists contains articles on many of the points above.

Software and code

Policy information about availability of computer code

Data collection	Ex-vivo electrophysiological data were collected using pClamp 10 (Axon Instrument). In-vivo electrophysiological data were collected using Open-Ephys GUI (v0.4), software that is publicly available (http://www.open-ephys.org/gui). Behavioral experiments were recorded using Ethovision XT 11.5 (Noldus).
Data analysis	Data analysis was performed using Clampfit 10.7 (Molecular Devices, USA), Mini analysis 6 (Synaptosoft, USA), Avisoft-SASlab Pro 5.0 (Avisoft Bioacustic, Germany), Offline Sorter (Plexon, USA), and Neuroexplorer 3 (Nex Technologies, USA). Statistical analysis was performed using SigmaPlot 11 (Systat, USA), GraphPad Prism 7.05 (GraphPad Software, San Diego, California, USA), and custom scripts written in MATLAB R2015a (MathWorks, USA). Experimental schemes and figures were created in Adobe Illustrator (v27.9).<br>The codes used for analysis have been deposited through Zenodo and are referenced in the manuscript.

For manuscripts utilizing custom algorithms or software that are central to the research but not yet described in published literature, software must be made available to editors and reviewers. We strongly encourage code deposition in a community repository (e.g. GitHub). See the Nature Portfolio guidelines for submitting code & software for further information.

Data

Policy information about availability of data

All manuscripts must include a data availability statement. This statement should provide the following information, where applicable:

- Accession codes, unique identifiers, or web links for publicly available datasets
- A description of any restrictions on data availability
- For clinical datasets or third party data, please ensure that the statement adheres to our policy

Source Data are provided with this paper. The document 'Source Data' includes all plotted data in individual Excel sheets. The data generated in this study have been deposited in the figshare database under accession code https://doi.org/10.6084/m9.figshare.30186424.
In addition, we provide the following Supplementary Data with this publication: 'Supplementary Data 1' includes statistical test results and details for all performed tests, divided on individual Excel sheets per Figure. The statistical tests performed with all plotted data in this study have been deposited in the zenodo database under accession code https://doi.org/10.5281/zenodo.17903734.

Research involving human participants, their data, or biological material

Policy information about studies with human participants or human data. See also policy information about sex, gender (identity/presentation), and sexual orientation and race, ethnicity and racism.

Reporting on sex and gender	*Use the terms sex (biological attribute) and gender (shaped by social and cultural circumstances) carefully in order to avoid confusing both terms. Indicate if findings apply to only one sex or gender; describe whether sex and gender were considered in study design; whether sex and/or gender was determined based on self-reporting or assigned and methods used. Provide in the source data disaggregated sex and gender data, where this information has been collected, and if consent has been obtained for sharing of individual-level data; provide overall numbers in this Reporting Summary. Please state if this information has not been collected. Report sex- and gender-based analyses where performed, justify reasons for lack of sex- and gender-based analysis.*
Reporting on race, ethnicity, or other socially relevant groupings	*Please specify the socially constructed or socially relevant categorization variable(s) used in your manuscript and explain why they were used. Please note that such variables should not be used as proxies for other socially constructed/relevant variables (for example, race or ethnicity should not be used as a proxy for socioeconomic status). Provide clear definitions of the relevant terms used, how they were provided (by the participants/respondents, the researchers, or third parties), and the method(s) used to classify people into the different categories (e.g. self-report, census or administrative data, social media data, etc.) Please provide details about how you controlled for confounding variables in your analyses.*
Population characteristics	*Describe the covariate-relevant population characteristics of the human research participants (e.g. age, genotypic information, past and current diagnosis and treatment categories). If you filled out the behavioural & social sciences study design questions and have nothing to add here, write "See above."*
Recruitment	*Describe how participants were recruited. Outline any potential self-selection bias or other biases that may be present and how these are likely to impact results.*
Ethics oversight	*Identify the organization(s) that approved the study protocol.*

Note that full information on the approval of the study protocol must also be provided in the manuscript.

Field-specific reporting

Please select the one below that is the best fit for your research. If you are not sure, read the appropriate sections before making your selection.

[x] Life sciences [ ] Behavioural & social sciences [ ] Ecological, evolutionary & environmental sciences

For a reference copy of the document with all sections, see nature.com/documents/nr-reporting-summary-flat.pdf

Life sciences study design

All studies must disclose on these points even when the disclosure is negative.

Sample size

No formal a priori statistical power calculation was performed. Sample sizes were determined based on established standards in systems neuroscience, prior experience with similar experimental paradigms, and extensive methodological expertise in oxytocin circuit research. Across all experimental modalities, effect sizes were sufficiently large to achieve robust statistical significance across independent experiments.
Anatomical and molecular experiments (immunohistochemistry, viral labeling and tracing, RNAscope in situ hybridization) typically included 3–5 animals per group, with multiple sections per animal analyzed. This approach is standard for circuit-mapping and gene-expression studies, where within-animal replication increases robustness and effect sizes are typically large.
Ex vivo experiments (electrophysiology and GRAB-OT sensor recordings) used cells or sections as the unit of analysis, obtained from multiple animals (typically 3–10 rats). Sample sizes of ~7–14 cells or ~10 sections per condition are commonly used in cellular physiology and neuropeptide release studies, with replication across animals explicitly verified.
Modulation experiments (optogenetics, chemogenetics, CRISPR/Cas9-mediated OTR knockdown) used 5–11 animals per group, consistent

with established practice for circuit manipulation and behavioral assays.
In vivo imaging experiments were performed in 4 animals, combined with large numbers of within-animal events, following a hierarchical design widely used in systems neuroscience.

Data exclusions	Data obtained from a total 15 rats across experiments were excluded from the analysis due to mistargeting or insufficient expression of viral vectors.
Replication	Experiments were repeated so that our data are based on at least three independent experiments with similar results. All attempts at replication were successful. The precise number of repeats are given in the text or in figure legends.
Randomization	Randomization was used to assign brain samples and animals to experimental groups whenever possible, with the constraint that in social behavior experiments rats had to be between unknown conspecifics, as described in Method section.
Blinding	Most of the measurements are made using a machine, and are not subject to operator bias, with the exception of manual scoring of social behaviors from videos; in this case, all scoring were done by two independent researchers (different from the one who performed the experiment) that was blind to treatment conditions.

Reporting for specific materials, systems and methods

We require information from authors about some types of materials, experimental systems and methods used in many studies. Here, indicate whether each material, system or method listed is relevant to your study. If you are not sure if a list item applies to your research, read the appropriate section before selecting a response.

Materials & experimental systems

n/a	Involved in the study
☐	☒ Antibodies
☐	☒ Eukaryotic cell lines
☒	☐ Palaeontology and archaeology
☐	☒ Animals and other organisms
☒	☐ Clinical data
☒	☐ Dual use research of concern
☒	☐ Plants

Methods

n/a	Involved in the study
☒	☐ ChIP-seq
☒	☐ Flow cytometry
☒	☐ MRI-based neuroimaging

Antibodies

Antibodies used

primary antibodies:
anti-OT (mouse, 1:2000, gift of H. Gainer, clone: PS38),
anti-RFP (Rockland 600-401-379, rabbit, 1:2000, polyclonal),
anti-GFP (Abcam ab13970, chicken, 1:1000, polyclonal),
anti-cFos (Cell Signaling 2250, rabbit, 1:500, clone: 9F6),
anti-NeuN (Merck MAB377, mouse, 1:1000, clone: A60),
anti-NeuN (Abcam ab177487, rabbit, 1:2000, clone: EPR12763),
anti-Ankyrin (Millipore MABN466, mouse, 1:1000, clone: N106/36),
anti-Parvalbumin (Abcam ab11427, rabbit, 1:5000, polyclonal),
anti-Somatostatin (BMA T-4103, rabbit, 1:3000, polyclonal),
anti-Calbindin (SWANT 300, mouse, 1:3000, clone: CB300),
and anti-Calretinin (SWANT 6B3, mouse, 1:5000 clone: 6B3).

secondary F(ab')$_2$ fragment donkey antibodies (Jackson ImmunoResearch, 1:1000, polyclonal)
anti-mouse Alexa 488-conjugated (715-546-150)
anti-chicken Alexa 488-conjugated (703-546-155)
anti-mouse Cy3-conjugated (715-166-150)
anti-rabbit Cy3-conjugated (711-166-152)
anti-mouse Alexa 647-conjugated (715-606-150)
anti-rabbit Alexa 647-conjugated (711-606-152)

Validation

Validation of anti-oxytocin (PS38) immunohistochemistry in rat was first reported in (Ben-Barak et al., J. of Neurosci., 1985). Validation of anti-GFP (Abcam ab13970) and anti c-Fos (Cell Signaling 2250) immunohistochemistry in rat was reported in (Hasan et al., Neuron, 2019). Additional information on the validation of all commercially available antibodies can be found on the respective manufacturers' catalogues.

Eukaryotic cell lines

Policy information about cell lines and Sex and Gender in Research

Cell line source(s)	Human embryonic kidney (HEK) 293T cell line was originally purchased from Addgene, USA (catalog number #240073).

Authentication	Cells authentication was done by microscopic inspection.
Mycoplasma contamination	All cell lines were tested negative for mycoplasma contamination.
Commonly misidentified lines (See ICLAC register)	No commonly misindentified cell lines were used in this study.

Animals and other research organisms

Policy information about studies involving animals; ARRIVE guidelines recommended for reporting animal research, and Sex and Gender in Research

Laboratory animals	Female Sprague-Dawley rats (3–8 weeks, Janvier Labs) and transgenic OTR-Cre rats bred in-house with a Sprague-Dawley background (Central Institute for Mental Health, Mannheim) were used. Adult virgin females (8–16 weeks) were included in anatomical, functional, and behavioral experiments. Animals were housed under standard laboratory conditions: 12-h light/dark cycle, lights on at 07:00, 22-24 °C, 50 +/- 5% humidity, free access to food and water.
Wild animals	The study did not involve wild animals.
Reporting on sex	The study was conducted with only female rats. Only female rats were used for this study, as male rats occasionally exhibit aggressive behaviors, which were out of scope for this study.
Field-collected samples	The study did not involve samples collected from the field.
Ethics oversight	All experiments were conducted under animal lisence G-193/20 and G-55/23 authorized by the German Animal Ethics Committee of the Baden Württemberg (Regierungspräsidium Karlsruhe, Germany) and in accordance with the German law, under license 3668-2016011815445431 from the French Ministry, and under EU regulations.

Note that full information on the approval of the study protocol must also be provided in the manuscript.

Plants

Seed stocks	*Report on the source of all seed stocks or other plant material used. If applicable, state the seed stock centre and catalogue number. If plant specimens were collected from the field, describe the collection location, date and sampling procedures.*
Novel plant genotypes	*Describe the methods by which all novel plant genotypes were produced. This includes those generated by transgenic approaches, gene editing, chemical/radiation-based mutagenesis and hybridization. For transgenic lines, describe the transformation method, the number of independent lines analyzed and the generation upon which experiments were performed. For gene-edited lines, describe the editor used, the endogenous sequence targeted for editing, the targeting guide RNA sequence (if applicable) and how the editor was applied.*
Authentication	*Describe any authentication procedures for each seed stock used or novel genotype generated. Describe any experiments used to assess the effect of a mutation and, where applicable, how potential secondary effects (e.g. second site T-DNA insertions, mosiacism, off-target gene editing) were examined.*
